# Network Analysis and Mediation Effect Analysis of Anxiety Symptoms and Sleep Patterns for Adolescents

**DOI:** 10.62641/aep.v53i5.1961

**Published:** 2025-10-05

**Authors:** Qiang He, Leilei Wang, Xingjie Yang, Yuchun Yang, Xuexue Meng, Libin Zhang, Xianyun Li, Jingxu Chen, Hu Deng, Wei Zheng, Wei Li, Yanping Shu, Xue Xiao

**Affiliations:** ^1^Department of Psychiatry of Women and Children, The Second People’s Hospital of Guizhou Province, 550004 Guiyang, Guizhou, China; ^2^Beijing HuiLongGuan Hospital, Peking University HuiLongGuan Clinical Medical School, 100096 Beijing, China; ^3^Faculty of Education, Beijing Normal University, 100875 Beijing, China; ^4^School of Psychology and Mental Health, North China University of Science and Technology, 066008 Qinhuangdao, Hebei, China; ^5^Collaborative Innovation Center of Assessment for Basic Education Quality, Beijing Normal University, 100875 Beijing, China; ^6^The Affiliated Brain Hospital, Guangzhou Medical University, 510370 Guangzhou, Guangdong, China; ^7^Key Laboratory of Neurogenetics and Channelopathies of Guangdong Province and the Ministry of Education of China, Guangzhou Medical University, 511436 Guangzhou, Guangdong, China; ^8^Department of Psychiatry, Beijing Tsinghua Changgung Hospital, School of Clinical Medicine, Tsinghua Medicine, Tsinghua University, 102218 Beijing, China

**Keywords:** anxiety symptom, sleep pattern, fatigue symptoms, network analysis, adolescents

## Abstract

**Background:**

The escalating prevalence of mental health issues and sleep disturbances among Chinese adolescents has become a pressing public health concern. Despite increasing recognition of this issue, there remains a paucity of research elucidating the intricate interplay between anxiety symptoms and sleep patterns. To address this critical gap, this study employs advanced network analysis to explore the complex relationships between these two domains, offering novel insights into their interconnectedness.

**Methods:**

We conducted a large-scale, online, cross-sectional survey encompassing 48,074 adolescents. Anxiety symptoms were assessed using the Generalised Anxiety Disorder Scale-7 (GAD-7), while sleep quality was evaluated via the Pittsburgh Sleep Quality Index (PSQI). Daytime sleepiness and fatigue symptoms were measured using the Chinese Adolescent Daytime Sleepiness Scale and the Fatigue Symptom Scale (FSS). Demographic data were analysed using chi-square tests, and continuous variables were examined via *t*-tests. To investigate symptom-level relationships, we employed network and mediation effect analysis, providing a robust methodological framework.

**Results:**

Our findings reveal a concerning prevalence of anxiety and insomnia symptoms among adolescents, at rates of 32.1% and 39.9%, respectively. Adolescents exhibiting anxiety symptoms reported significantly higher levels of insomnia, fatigue, and daytime sleepiness. Mediation effect analysis uncovered a chain mediation pathway, whereby insomnia and fatigue symptoms sequentially mediated the relationship between anxiety and daytime sleepiness. Network analysis further identified GAD2 (uncontrollable worry) and PSQI7 (daytime function) as pivotal core symptoms, with FSS (fatigue symptoms) demonstrating the highest expected impact within the network.

**Conclusions:**

This study underscores the persistent severity of anxiety and sleep pattern disturbances among adolescents. These findings highlight the importance of addressing core symptoms—particularly GAD2 and PSQI7—in therapeutic interventions. The results provide a solid foundation for developing more effective, symptom-specific strategies to improve adolescent mental health and sleep quality.

## Introduction

Adolescence represents a pivotal stage of human development, characterised by profound physical, psychological, and social transformations. During this period, anxiety has emerged as a prevalent mental health concern. Recent studies indicate a worrying upward trend in adolescent anxiety rates, with prevalence among American adolescents doubling from approximately 10% to 20% between 2016 and 2020 [1, 2, 3]. Similarly, European nations have witnessed a surge in anxiety cases, with reported rates ranging from 15% to 25% in countries such as the United Kingdom and Germany [4, 5, 6]. The situation appears particularly acute in China, where adolescent anxiety rates have escalated from 10% to over 20% in the past decade, with some studies reporting prevalence exceeding 40% [7, 8, 9, 10]. Without timely intervention, these anxiety symptoms risk progressing into full-blown anxiety disorders, which currently rank among the most prevalent mental health conditions in adolescents globally. Notably, anxiety disorders are the third and fourth leading causes of years lost due to disability for individuals aged 15–19 and 20–24, respectively, underscoring their significant impact on adolescent health and well-being [11]. These alarming statistics highlight the critical need for focused attention and comprehensive research into adolescent anxiety.

Anxiety symptoms exhibit a strong correlation with insomnia symptoms [12]. Individuals experiencing anxiety often enter a state of hyperarousal, marked by heightened alertness, fear, and nervousness, which disrupts sleep initiation and maintenance. Physiological manifestations of anxiety, such as accelerated heart rate and sweating, combined with persistent negative cognitive patterns, further impede the brain’s ability to relax, exacerbating insomnia symptoms [13, 14, 15]. Research indicates that 60% to 70% of individuals with generalised anxiety disorder experience sleep disturbances, including prolonged sleep latency and reduced deep sleep [16]. Adolescent studies further reveal that anxiety is associated with diminished total sleep time, poor sleep quality, and insomnia symptoms [12, 17]. Additionally, the interplay between insomnia and anxiety often leads to daytime sleepiness, significantly impairing adolescents’ academic performance and daily functioning.

Fatigue, characterised by persistent physical and mental exhaustion unrelieved by rest, is another critical consequence of anxiety [18]. Excessive anxiety places adolescents in a prolonged state of hypervigilance, depleting their energy reserves and resulting in profound fatigue. Accompanying physical symptoms, such as muscle tension, body aches, and general weariness, further exacerbate this condition [19]. Daytime sleepiness is also prevalent among adolescents, with studies reporting that 25%–75% experience this issue. Daytime sleepiness is closely linked to subjective feelings of depression, anxiety, and sleep deprivation, adversely affecting adolescents’ daytime activities and overall well-being [20].

To explore in depth the complex interaction between anxiety symptoms in adolescents and insomnia, fatigue, and daytime sleepiness, this study adopts network analysis and treats these symptoms as a dynamic, interactive system. Unlike the traditional linear research paradigm, this method not only visually presents the correlation network among various symptoms, but also accurately quantifies the intensity of their direct and indirect interactions and identifies the key symptom nodes at the core of the network—namely, the hub symptoms that exert significant influence over other symptoms. These nodes often represent the most valuable targets for clinical intervention [21, 22, 23]. At present, network analysis is also applied to the analysis, diagnosis, and treatment of mental disorders. Building upon this approach, the current study further employs mediation effect analysis to systematically examine the internal pathways through which these symptoms influence one another and to explore their potential pathogenesis. The combined use of these two analytical methods facilitates a comprehensive understanding of the overall symptom network configuration and provides a precise basis for clinical intervention.

In summary, the rising prevalence of anxiety symptoms among adolescents underscores the importance of investigating their intricate relationships with insomnia, fatigue, and daytime sleepiness. Despite the growing global recognition of these issues, research in China remains limited. This study employs a large-scale, cross-sectional design involving five provinces in China to examine the incidence of anxiety symptoms in adolescents. By utilising advanced network analysis and mediation effect analysis, it aims to elucidate the complex interrelationships among these symptoms, offering valuable insights for targeted interventions and improved mental health outcomes.

## Methods

### Study Design and Participants

This study employed a cross-sectional design, conducted from 9 January to 20 January 2023, targeting adolescents aged 12 to 20 years. Participants were recruited via the Wenjuanxing platform (https://www.wjx.cn/app/survey.aspx), with a primary focus on junior high and senior high school students from five provinces and autonomous regions in China: Shandong, Guangxi, Inner Mongolia, Xinjiang, and Hebei. A total of 52,964 participants initially completed the survey. School teachers distributed the Wenjuanxing QR code to class groups, and students completed the questionnaire voluntarily and anonymously using their parents’ mobile phones. The inclusion criteria for this study were as follows: (1) junior high or senior high school student status; (2) ability to read and comprehend Chinese questionnaires; and (3) Chinese nationality. The exclusion criteria included: (1) incomplete or partially completed questionnaires and (2) response time of less than 300 seconds, which indicated a high likelihood of random answering. To ensure data integrity, rigorous quality control measures were applied, resulting in the exclusion of 4890 questionnaires and yielding a final response rate of 91.8%.

The study protocol received ethical approval from the Ethics Committee of Beijing Huilongguan Hospital (approval id: 2022-87- Scientific Research). The study was conducted in accordance with the ethical standards outlined in the Declaration of Helsinki. Participation was entirely voluntary, and informed consent was obtained from both participants and their legal guardians prior to enrolment. This approach ensured adherence to ethical standards and protected the rights and confidentiality of all participants.

### Assessment Tools and Procedure

#### Sociodemographic Factors

Demographic data were collected to capture key sociodemographic variables, including grade level, gender, age, place of residence (urban vs. rural), and household composition (e.g., living with parents, grandparents, or others).

#### Anxiety Symptoms

The Generalised Anxiety Disorder Scale-7 (GAD-7) was used to assess anxiety symptoms experienced by adolescents over the preceding two weeks. The scale comprises seven items, each rated on a 4-point Likert scale ranging from 0 to 3, yielding a total score from 0 to 21. Scores were categorised as follows: 0–4 (no anxiety), 5–9 (mild anxiety), 10–14 (moderate anxiety), and 15–21 (severe anxiety) [24]. The GAD-7 has demonstrated high reliability and validity in previous studies, and in this study, it exhibited excellent internal consistency, with a Cronbach’s α coefficient of 0.945.

#### Sleep Quality

Sleep quality was evaluated using the Pittsburgh Sleep Quality Index (PSQI), a widely validated instrument comprising 19 items grouped into seven components: subjective sleep quality, sleep latency, sleep duration, habitual sleep efficiency, sleep disturbances, use of sleep medication, and daytime dysfunction. Each component is scored on a scale from 0 to 3, where 0 indicates no difficulty, 1 indicates mild difficulty, 2 indicates moderate difficulty, and 3 indicates severe difficulty. In this study, each component was considered abnormal if its score was greater than 0. The total PSQI score, obtained by summing the seven component scores, ranges from 0 to 21, with higher scores reflecting poorer sleep quality. A total score greater than 5 was considered indicative of insomnia symptoms [25]. In this study, the PSQI demonstrated good reliability, with a Cronbach’s α coefficient of 0.705.

#### Daytime Sleepiness

Daytime sleepiness was measured using the Chinese Adolescent Daytime Sleepiness Scale (CADSS), which comprises seven items rated on a 5-point Likert scale (1–5). Total scores range from 7 to 35, with classifications as follows: <16 (normal), 17–22 (mild), 23–30 (moderate), and 31–35 (severe daytime sleepiness) [26]. The CADSS demonstrated high internal consistency in this study, with a Cronbach’s α of 0.921.

#### Fatigue

Fatigue severity was assessed using the Fatigue Severity Scale (FSS), a nine-item instrument rated on a 7-point Likert scale (1 = “strongly disagree” to 7 = “strongly agree”). The average score was used to determine fatigue severity, with scores categorised as <4 (no fatigue), 4–4.9 (moderate fatigue), and ≥5 (severe fatigue) [27]. The FSS exhibited excellent reliability in this study, with a Cronbach’s α of 0.932.

These validated instruments collectively provided a comprehensive assessment of key psychological and physiological constructs, ensuring robust measurement of anxiety, sleep quality, daytime sleepiness, and fatigue among the adolescent participants.

#### Statistical Analysis

IBM SPSS Statistics version 26.0 (Chicago, IL, USA) and JASP version 0.19.0.0 (Amsterdam, University of Amsterdam, Netherlands) were used for the data analysis. The chi-square test was used to compare the effects of demographic variables on anxiety symptoms and to compare differences in insomnia symptoms between adolescents with and without anxiety. Pearson correlation analysis was conducted to examine the relationships among anxiety symptoms, insomnia symptoms, daytime sleepiness, and fatigue. The level of statistical significance was set at *p* < 0.05.

We used Model 6 of the PROCESS macro (version 3.4) for SPSS 26.0 to analyse the chain mediation effect and explore the roles of anxiety symptoms, insomnia symptoms, fatigue symptoms, and daytime sleepiness. Anxiety symptoms were designated as the independent variable, fatigue symptoms as the dependent variable, and insomnia symptoms and daytime sleepiness as the mediating variables. The output indicators included the total effect, direct effect, and indirect effect. The bootstrap method was employed to test the mediation effects, with the sample size set at 5000 and a 95% confidence interval (CI) selected for both direct and indirect effects. An indirect effect was considered statistically significant if its 95% CI did not contain zero.

Network analysis was conducted using JASP version 0.19.0.0. A Graphical Gaussian Model was used to estimate the partial correlation network among symptoms, with nodes representing symptoms (e.g., anxiety, insomnia, daytime sleepiness, fatigue) and edges representing their partial correlations [21, 22]. The matrix was constructed using Pearson partial correlations, and the network was generated using graphical least absolute shrinkage and selection operator in combination with the extended Bayesian information criterion. In the visualisation, central nodes had stronger connections, with blue solid lines indicating positive correlations and red dotted lines indicating negative correlations [23]. Centrality indices were used to assess the influence of each symptom within the network. Strength refers to the absolute weight of the edges connected to a symptom, reflecting its potential to affect other symptoms. Closeness refers to the inverse of the shortest path between a symptom and all other symptoms—greater closeness indicates a higher degree of mutual influence. Betweenness refers to the frequency with which a symptom lies on the shortest path between two other symptoms, reflecting its role as a bridge in the network. Expected impact refers to the average extent to which a node may influence others in the network; it comprehensively reflects direct connections, network position, and the manner in which influence propagates. Edge weight accuracy was assessed using non-parametric bootstrapping (1000 iterations), and network stability was tested using case-dropping bootstrap procedures.

## Results

### Sociodemographic Characteristics and Their Relationship with Anxiety Symptoms

The study included 48,074 adolescents with a mean age of 16.86 ± 1.70 years. Among them, 23,443 (48.8%) were middle school students and 24,631 (51.2%) were high school students. The sample comprised 22,486 (46.8%) males and 25,588 (53.2%) females. A total of 15,408 adolescents exhibited anxiety symptoms, resulting in an incidence rate of 32.1%. Anxiety symptoms were more prevalent among females than males (37.2% vs. 26.2%) and among senior high school students compared to junior high school students (38.1% vs. 25.6%). Adolescents living with grandparents exhibited a higher incidence of anxiety symptoms than those living with parents (39.8% vs. 31.8%). Additionally, anxiety symptoms were more common in adolescents from hometown than in those from city (32.9% vs. 31.5%) (Table 1).

**Table 1.** **Socio-demographic characteristics and associations with anxiety symptom group (N = 48,074)**.

Variables		All	With anxiety symptom	Without anxiety symptom	χ ^2^	*Phi*	*p*
		N (%)	n (%)	n (%)			
Gender					672.43	0.12	<0.001
	Male	22,486 (46.8)	5883 (26.2)	16,603 (73.8)			
	Female	25,588 (53.2)	9525 (37.2)	16,063 (62.8)			
Grade					860.86	0.13	<0.001
	Junior high school	23,443 (48.8)	6013 (25.6)	17,430 (74.4)			
	Senior high school	24,631 (51.2)	9395 (38.1)	15,236 (61.9)			
Live together					42.13	0.03	<0.001
	Parents	46,597 (96.9)	14,820 (31.8)	31,777 (68.2)			
	Grandparents	1477 (3.1)	588 (39.8)	889 (60.2)			
Residence					10.14	0.02	0.001
	City	29,260 (60.9)	9219 (31.5)	20,041 (68.5)			
	Hometown	18,814 (30.1)	6189 (32.9)	12,625 (67.1)			

### Insomnia Symptoms and Sleep Patterns of Adolescents With or Without Anxiety Symptoms

The study revealed that 39.9% of adolescents experienced insomnia symptoms, 48.9% reported fatigue symptoms, and 35.9% exhibited symptoms of daytime sleepiness. Adolescents with anxiety symptoms demonstrated a significantly higher incidence of insomnia compared to those without anxiety (73.2% vs. 24.2%). They also reported poorer subjective sleep quality, longer sleep latency, shorter sleep duration, lower sleep efficiency, and later bedtimes. Among insomnia symptoms, sleep disturbances were the most prevalent (73.5%), followed by daytime dysfunction (64.5%), with both being more pronounced in adolescents with anxiety. Additionally, the use of sleep medication was more frequent among adolescents with anxiety symptoms. Furthermore, anxious adolescents exhibited higher rates of fatigue and daytime sleepiness than their non-anxious peers (Table 2).

**Table 2.** **The incidence rate and patterns of insomnia symptoms for with or without anxiety symptom group (N = 48,074)**.

Variables		All	With anxiety symptom	Without anxiety symptom	χ ^2^	*Phi/Cramer V*	*p*
		N (%)	n (%)	n (%)			
Insomnia		19,195 (39.9)	11,286 (73.2)	7909 (24.2)	10,495.70	0.48	<0.001
Subjective sleep quality					9977.50	0.46	<0.001
	Excellent	16,008 (33.3)	1491 (9.7)	14,517 (44.4)			
	Good	22,904 (47.6)	7517 (48.8)	15,387 (47.1)			
	Bad	7571 (15.7)	5075 (32.9)	2476 (7.6)			
	Poor	1591 (3.3)	1325 (8.6)	266 (0.8)			
Sleep latency					1917.72	0.20	
	≤15 minutes	23,760 (49.4)	5910 (38.4)	17,850 (54.8)			
	16–30 minutes	19,623 (40.1)	6855 (44.5)	12,768 (39.1)			
	31–60 minutes	3749 (7.8)	2072 (13.4)	1677 (5.1)			
	≥61 minutes	942 (2.0)	571 (3.7)	371 (1.1)			
Sleep duration (hours)					2537.27	0.23	<0.001
	>7	28,006 (58.3)	6702 (43.5)	21,304 (65.2)			
	6–7	11,334 (23.6)	4185 (27.2)	7149 (21.9)			
	5–6	7929 (16.5)	4138 (26.9)	3790 (11.6)			
	<5	806 (1.7)	383 (2.5)	423 (1.3)			
Sleep efficiency					154.76	0.06	<0.001
	>85%	33,307 (39.2)	10,155 (21.2)	23,152 (48.2)			
	75%∼84%	8328 (17.3)	2834 (18.4)	5459 (16.8)			
	65%∼74%	3309 (6.9)	1177 (7.6)	2132 (6.5)			
	<65%	3130 (6.5)	1242 (8.1)	1888 (5.8)			
Bedtime					1725.61	0.19	<0.001
	Before 22 o’clock	5441 (11.3)	1264 (8.2)	4177 (12.8)			
	Greater than or equal to 22 o’clock and less than 24	27,662 (57.5)	7392 (48.0)	20,270 (62.1)			
	No less than 24 o’clock	14,971 (31.1)	6572 (43.8)	8219 (25.2)			
Sleep disturbance		35,355 (73.5)	14,297 (92.8)	21,058 (64.5)	4317.03	0.30	<0.001
Used sleep medication		1137 (2.4)	909 (5.9)	228 (0.7)	1226.72	0.16	<0.001
Daytime dysfunction		31,437 (65.4)	14,423 (93.6)	17,014 (52.1)	7976.33	0.41	<0.001
Fatigue symptom		23,488 (48.9)	10,667 (69.2)	12,821 (39.2)	3766.39	0.28	<0.001
Daytime sleepiness					8776.91	0.43	<0.001
	Normal	30,812 (64.1)	5530 (35.9)	25,282 (77.4)			
	Mild	10,541 (21.9)	5162 (33.5)	5379 (16.5)			
	Moderate	5023 (10.4)	3364 (21.8)	1659 (5.1)			
	Severe	1698 (3.5)	1352 (8.8)	346 (1.1)			

### The Relationship Between Anxiety Symptoms and Insomnia, Fatigue, and Daytime Sleepiness

**Fig. 1.**
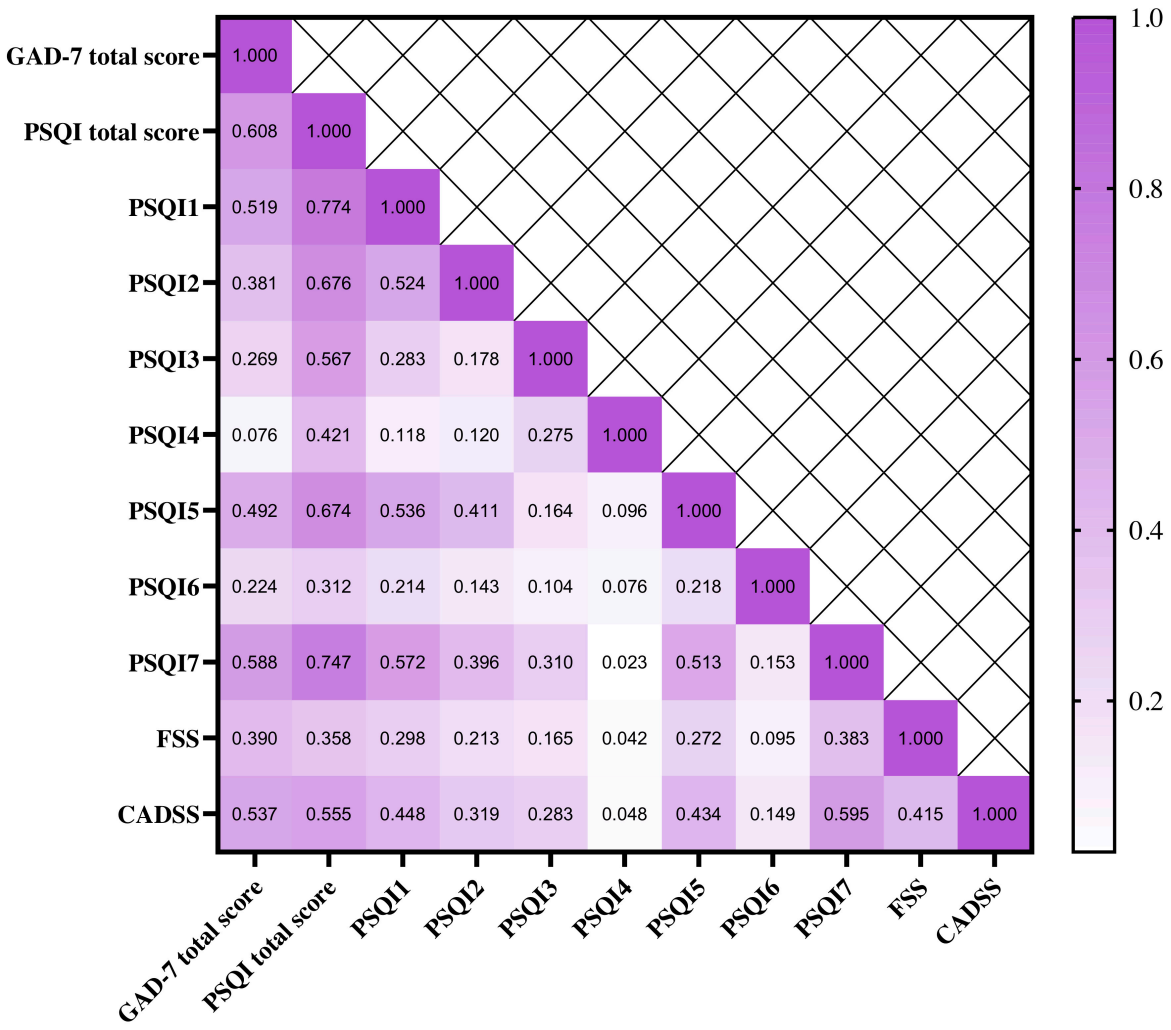
**Pearson’s correlation analysis between anxiety, insomnia, fatigue symptoms and daytime sleepiness**. PSQI1, Subjective sleep quality; PSQI2, Sleep latency; PSQI3, Sleep duration; PSQI4, Sleep efficiency; PSQI5, Sleep disturbance; PSQI6, Used sleep medication; PSQI7, Daytime dysfunction; CADSS, Chinese Adolescent Daytime Sleepiness Questionnaire; FSS, Fatigue Severity Scale; GAD-7, Generalized Anxiety Disorder Scale-7; PSQI, Pittsburgh Sleep Quality Index questionnaire. Note: all *p* values < 0.001.

Correlation analysis revealed significant pairwise positive correlations between anxiety symptoms, total sleep symptom scores, individual sleep components, fatigue symptoms, and daytime sleepiness (all *p* < 0.001) (Fig. 1).

### The Chain Mediation Effect of Insomnia and Fatigue Symptoms Between Anxiety and Daytime Sleepiness

**Fig. 2.**
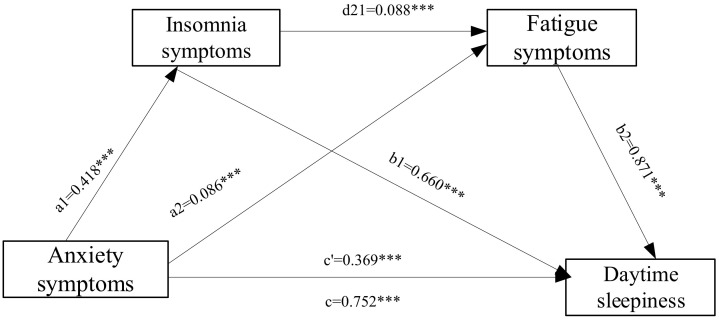
**The role of insomnia symptoms and fatigue symptoms in the chain mediation of anxiety symptoms and Daytime sleepiness**. Note: a1 represents the effect value of anxiety symptoms positively predicting insomnia symptom; a2 represents the effect value by which anxiety symptoms positively predict fatigue symptoms; b1 represents the positive predictive effect value of insomnia symptoms on daytime sleepiness; b2 represents the effect value of the positive prediction of fatigue symptoms on daytime sleepiness; c’ represent direct effect; c represents the total effect; d21 represents the effect value of insomnia symptoms positively predicting fatigue symptoms. ***: *p* < 0.001.

Chain mediation analysis demonstrated that the direct effect of anxiety symptoms on daytime sleepiness was 0.369, accounting for 49.04% of the total effect. The 95% confidence intervals for all indirect effects excluded zero, confirming their statistical significance. The total indirect effect was 0.383, accounting for 50.95% of the total effect. The three specific mediation pathways were as follows: path 1 = 0.276 (36.67%), path 2 = 0.075 (9.98%), and path 3 = 0.032 (4.25%). The total effect was 0.752 (Table 3 and Fig. 2).

**Table 3.** **The role of insomnia and fatigue symptoms in the chain mediation of anxiety symptoms and daytime sleepiness (N = 48,074)**.

Mediation model	Mediation path	Effect (β)	Standard error (SE)	The proportion of mediation effect (%)	95% confidence interval (CI)	
					Lower	Upper
Indirect effect	Path 1: X→M1→Y	0.276	0.005	36.67	0.267	0.285
	Path 2: X→M2→Y	0.075	0.002	9.98	0.071	0.079
	Path 3: X→M1→M2→Y	0.032	0.001	4.25	0.030	0.034
	Total indirect effect	0.383	0.005	50.95	0.373	0.393
Direct effect		0.369	0.006	49.04	0.356	0.381
Total effect		0.752	0.005		0.741	0.762

Path 1: X→M1→Y: anxiety symptoms → insomnia symptoms → daytime sleepiness. Path 2: X→M2→Y: anxiety symptoms → fatigue symptoms → daytime sleepiness. Path 3: X→M1→M2→Y: anxiety symptoms → insomnia symptoms → fatigue symptoms → daytime sleepiness. X, anxiety symptoms; M1, anxiety symptoms; M2, fatigue symptoms; Y, daytime sleepiness.

### Network Analysis Among Anxiety, Insomnia, Fatigue Symptoms and Daytime Sleepiness

**Fig. 3.**
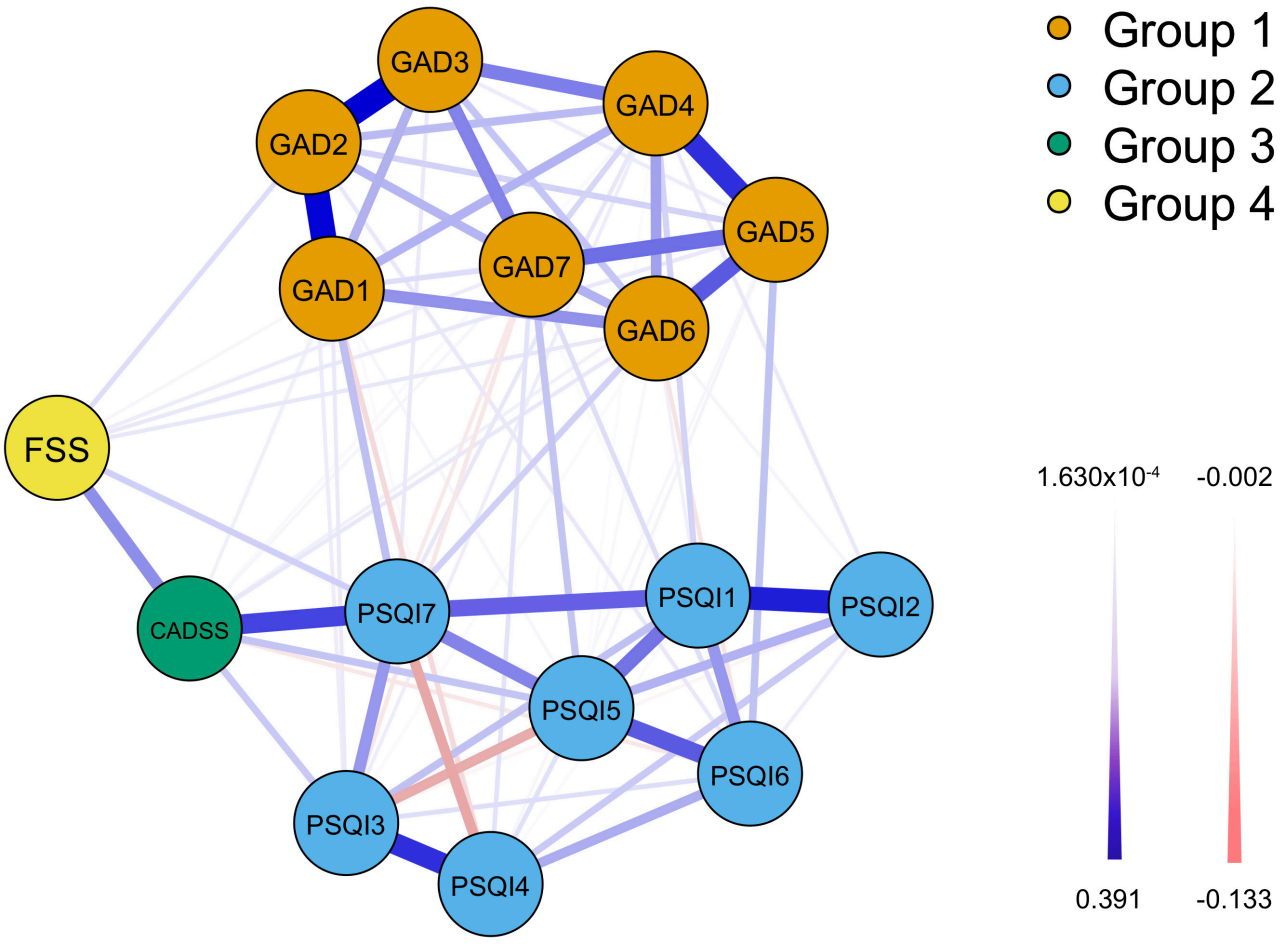
**A network analysis model of adolescent anxiety, insomnia, daytime sleepiness and fatigue symptoms**. Note: GAD1, Nervousness; GAD2, Uncontrollable worrying; GAD3, Worry too much; GAD4, Trouble relaxing; GAD5, Restlessness; GAD6, Irritability; GAD7, Feeling afraid; PSQI1, Subjective sleep quality; PSQI2, Sleep latency; PSQI3, Sleep duration; PSQI4, Sleep efficiency; PSQI5, Sleep disturbance; PSQI6, Used sleep medication; PSQI7, Daytime dysfunction; CADSS, Chinese Adolescent Daytime Sleepiness Questionnaire; FSS, Fatigue Severity Scale.

**Fig. 4.**
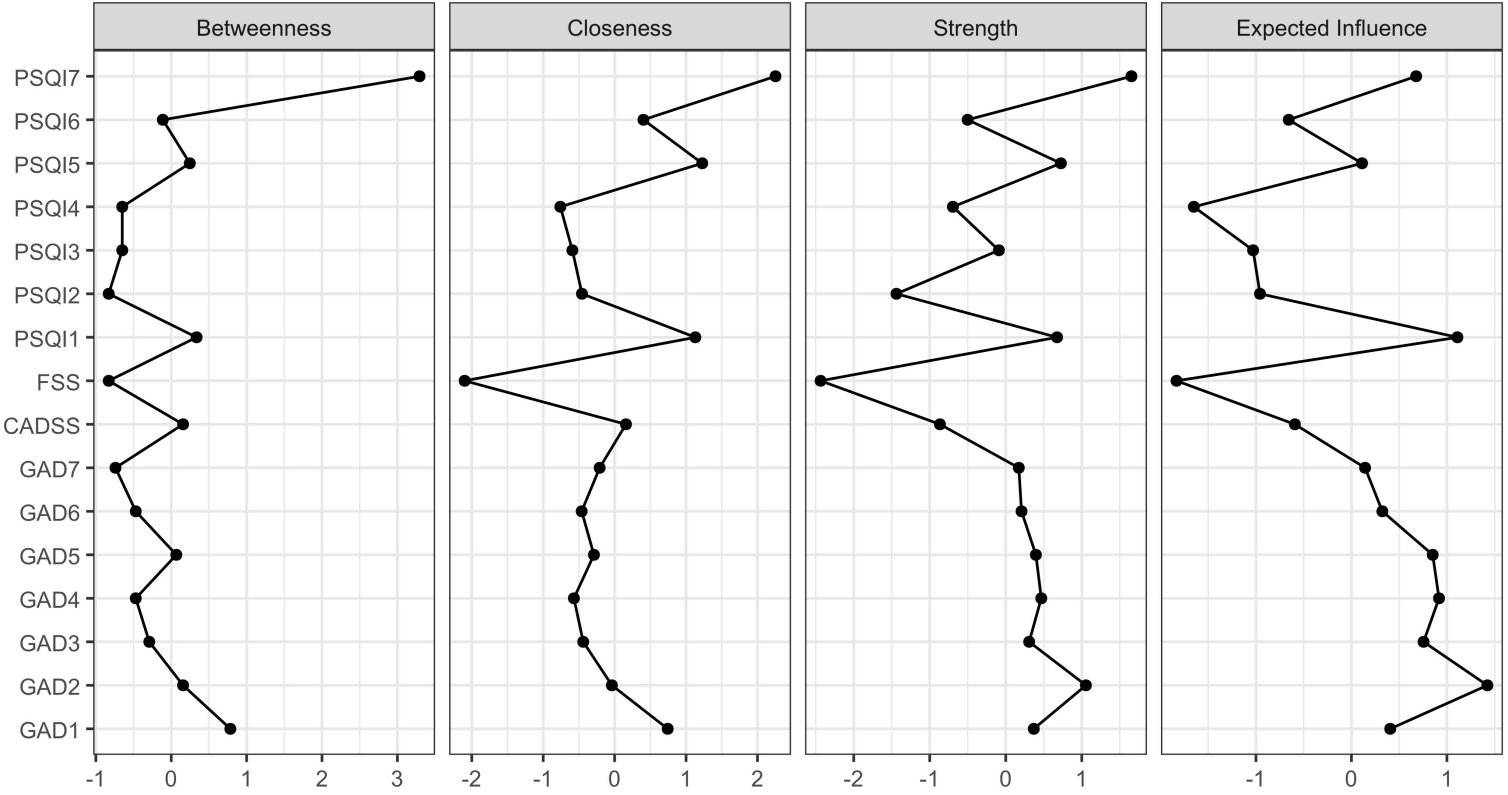
**Central indicators of each symptom in adolescents**. The X-axis represents the size of Z-scores, including Betweenness, Closeness, Strength and Expected Influence; and the Y-axis represents the names of the factors GAD-7, PSQI, FSS, and CADSS. Note: GAD1, Nervousness; GAD2, Uncontrollable worrying; GAD3, Worry too much; GAD4, Trouble relaxing; GAD5, Restlessness; GAD6, Irritability; GAD7, Feeling afraid; PSQI1, Subjective sleep quality; PSQI2, Sleep latency; PSQI3, Sleep duration; PSQI4, Sleep efficiency; PSQI5, Sleep disturbance; PSQI6, Used sleep medication; PSQI7, Daytime dysfunction; CADSS, Chinese Adolescent Daytime Sleepiness Questionnaire; FSS, Fatigue Severity Scale.

In the network analysis, centrality indicators revealed that among adolescents presenting with insomnia symptoms, PSQI7 (daytime dysfunction) exhibited the highest strength (1.653), betweenness centrality (3.294), and closeness centrality (2.253), identifying it as both a core and bridge symptom. The nodes PSQI1 (subjective sleep quality) and PSQI2 (sleep latency) demonstrated the strongest direct connection, followed by PSQI3 (sleep duration) and PSQI4 (sleep efficiency). PSQI4 also displayed the highest expected influence (–1.651). Similarly, among adolescents with anxiety symptoms, GAD2 (uncontrollable worrying) exhibited the highest strength (1.054), while GAD1 demonstrated the highest betweenness centrality (0.784), closeness centrality (0.743), and expected influence (1.424), indicating their roles as core and bridge symptoms within the anxiety network. Nodes GAD2 and GAD3 (worrying too much) showed the strongest connection, followed by GAD1 and GAD2. Across symptom domains, the most direct connections were observed between GAD1 and PSQI7, CADSS and PSQI7, and FSS and CADSS (Figs. 3,4).

The accuracy of edge weights is illustrated as follows: the black line represents the average edge weights estimated using the self-sampling method, while the red line represents the edge weights derived from the sample in this study. The grey area indicates the 95% confidence interval. The narrow 95% confidence interval for the edge weights in this study suggests a relatively precise estimation. The stability test for the node centrality indices showed that closeness, strength, and betweenness values were all above 0.75, indicating stable estimates of node centrality (**Supplementary Figs. 1,2**).

## Discussion

This cross-sectional study examined the prevalence of anxiety and insomnia symptoms among adolescents, as well as the impact of anxiety on sleep-related outcomes, including insomnia, fatigue, and daytime sleepiness. By employing network analysis and mediation effect analysis, we investigated the complex interrelationships among anxiety symptoms, insomnia, fatigue, and daytime sleepiness.

The results indicated that 32.1% of adolescents experienced anxiety symptoms, representing an increase compared to previous studies (9%–26%) [28, 29, 30]. This finding highlights anxiety as a significant and growing public health concern in this population. Persistent anxiety can profoundly affect psychological well-being, academic performance, social functioning, physical health, and behaviour, potentially resulting in diminished academic achievement, social withdrawal, interpersonal conflict, distorted self-image, and reduced immune function [31, 32, 33, 34]. Alarmingly, some adolescents adopt maladaptive coping strategies, including alcohol or drug use and non-suicidal self-injury, thereby increasing the risk of addictive behaviours [35, 36]. These findings underscore the urgent need for targeted interventions to mitigate adolescent anxiety and its broad-ranging consequences.

Our study revealed a higher prevalence of anxiety symptoms among girls compared to boys, consistent with previous research [37]. This gender disparity may be attributed to significant hormonal fluctuations in adolescent girls—particularly changes in oestrogen and progesterone levels—which affect emotional regulation and increase vulnerability to anxiety [38]. In addition, girls may exhibit heightened sensitivity in brain regions associated with emotional processing, such as the amygdala, leading to stronger stress responses [39]. Moreover, girls are generally more expressive about their emotions and more likely to report anxiety symptoms [40].

The study also found that anxiety symptoms were more pronounced among high school students, possibly due to intense academic pressure and the pivotal role of the gaokao, a high-stakes university entrance examination in China [41]. Furthermore, adolescents living with grandparents or residing in rural areas exhibited higher rates of anxiety symptoms. This may result from grandparents tending to prioritise physical health over mental well-being and the higher proportion of “left-behind” children in rural regions, who are known to experience elevated levels of anxiety and depression [42]. These findings underscore the need for targeted mental health interventions tailored to these particularly vulnerable groups.

Our study highlights the concerning state of sleep quality among Chinese adolescents, with a notable rise in the prevalence of insomnia, daytime sleepiness, and fatigue symptoms [43, 44, 45]. This decline may be attributed to hormonal fluctuations during adolescence, which disrupt the sleep–wake cycle. Adolescents’ natural tendency toward a nocturnal circadian rhythm often conflicts with school schedules, resulting in social jet lag [46, 47]. Furthermore, the transition to middle and high school imposes increased academic demands, contributing to sleep deprivation [48, 49]. Exacerbating the problem, many adolescents routinely use mobile phones and tablets before bedtime, exposing themselves to blue light that suppresses melatonin secretion and further disturbs sleep patterns [50].

In addition, the influence of psychological factors on adolescent sleep should not be overlooked. Our study found that anxiety symptoms significantly affect sleep, with the prevalence of insomnia symptoms being approximately three times higher in adolescents with anxiety than in those without. These individuals also exhibited pronounced sleep disturbances, including prolonged sleep latency, reduced sleep duration, and disrupted circadian rhythms. Anxiety symptoms further intensified fatigue and daytime sleepiness, as the persistent tension, worry, and somatic discomfort associated with anxiety led to both mental and physical exhaustion. This, in turn, impaired daytime functioning, contributing to sleepiness, diminished academic performance, and elevated anxiety levels—thereby perpetuating a vicious cycle [20, 51]. Our mediation effect analysis supports this pathway, indicating that anxiety-induced insomnia contributes to daytime fatigue, which subsequently results in daytime sleepiness. These findings underscore the complex interplay between anxiety, sleep disturbances, and daytime functioning in adolescents.

Previous research has demonstrated that network analysis is a powerful methodological framework for investigating complex psychopathological systems, offering distinct advantages over traditional statistical approaches in identifying core symptoms and elucidating their dynamic interactions [52]. Our study identified GAD2 as the most central symptom in the anxiety network, exhibiting the highest strength and expected influence. GAD1 and GAD3 were found to be most closely connected to GAD2, underscoring its pivotal role in the anxiety symptom network. This positions GAD2 as a critical target for intervention in adolescent anxiety. GAD2 reflects persistent and uncontrollable worry about perceived threats, indicating that adolescents may be trapped in a state of chronic, unmanageable anxiety. These findings are consistent with previous research on frontline caregivers, which similarly emphasises the centrality of uncontrollable worrying in anxiety disorders [53, 54]. Targeting GAD2 in therapeutic interventions may therefore offer a strategic approach to alleviating anxiety symptoms in adolescents.

In the context of insomnia symptoms, PSQI7 emerged as the most central node in the network analysis, highlighting its role as a core feature of adolescent insomnia. It also demonstrated the strongest associations with GAD1, FSS (fatigue symptoms), and CADSS (daytime sleepiness). Notably, the connection between FSS and GAD1 was particularly robust. Nervousness—characterised by tension, anxiety, or restlessness—can lead to persistent worry, irritability, difficulty relaxing, muscle tension, fatigue, sleep disturbances, and impaired concentration. This state of hyperarousal and physiological overactivation significantly disrupts both sleep patterns and daytime functioning, underscoring the intricate interrelationships among these symptoms in adolescents.

This study has several limitations. First, as it employed a cross-sectional design, it lacks longitudinal follow-up to track changes in anxiety and sleep patterns over time. Second, although we examined the associations between anxiety symptoms and sleep patterns, the underlying biological mechanisms were not explored. Third, reliance on self-reported measures introduces potential subjective bias, which may affect the accuracy of the assessments. Nevertheless, self-report instruments remain the most widely used tools for mental health evaluation due to their efficiency, convenience, and practicality. Future research should incorporate longitudinal designs, biological assessments, and multi-method approaches to address these limitations and provide a more comprehensive understanding of the complex interplay between anxiety and sleep disturbances.

## Conclusion

This study reveals a high prevalence of anxiety and insomnia symptoms among adolescents, with anxiety exerting a significant influence on sleep patterns. Network analysis identified GAD2 and PSQI7 as the most central symptoms, highlighting them as potential targets for intervention. Mediation analysis further demonstrated that insomnia and fatigue symptoms act as sequential mediators in the relationship between anxiety and daytime sleepiness. These findings advance our understanding of the intricate links between anxiety and sleep disturbances in adolescents. By employing advanced network analysis, we identified critical intervention points that may help to disrupt the cyclical relationship between anxiety and impaired sleep, offering a foundation for more effective prevention and treatment strategies in adolescent mental health care.

## Availability of Data and Materials

The datasets generated and/or analyzed during the current study are not publicly available but are available from the corresponding author upon reasonable request.

## Supplementary Material

Supplementary material associated with this article can be found, in the online version, at https://doi.org/10.62641/aep.v53i5.1961.
