# Supplementary material for: Network Analysis and Mediation Effect Analysis of Anxiety Symptoms and Sleep Patterns for Adolescents
Source: Actas Esp Psiquiatr. 2025 Oct 5;53(5):1040–52. doi: 10.62641/aep.v53i5.1961 (PMC12538618; doi:10.62641/aep.v53i5.1961)
Supplement: Supplementary file 1 [file ActEsp-53-5-1040-1052-s1.zip › Supplementary+materials.docx]

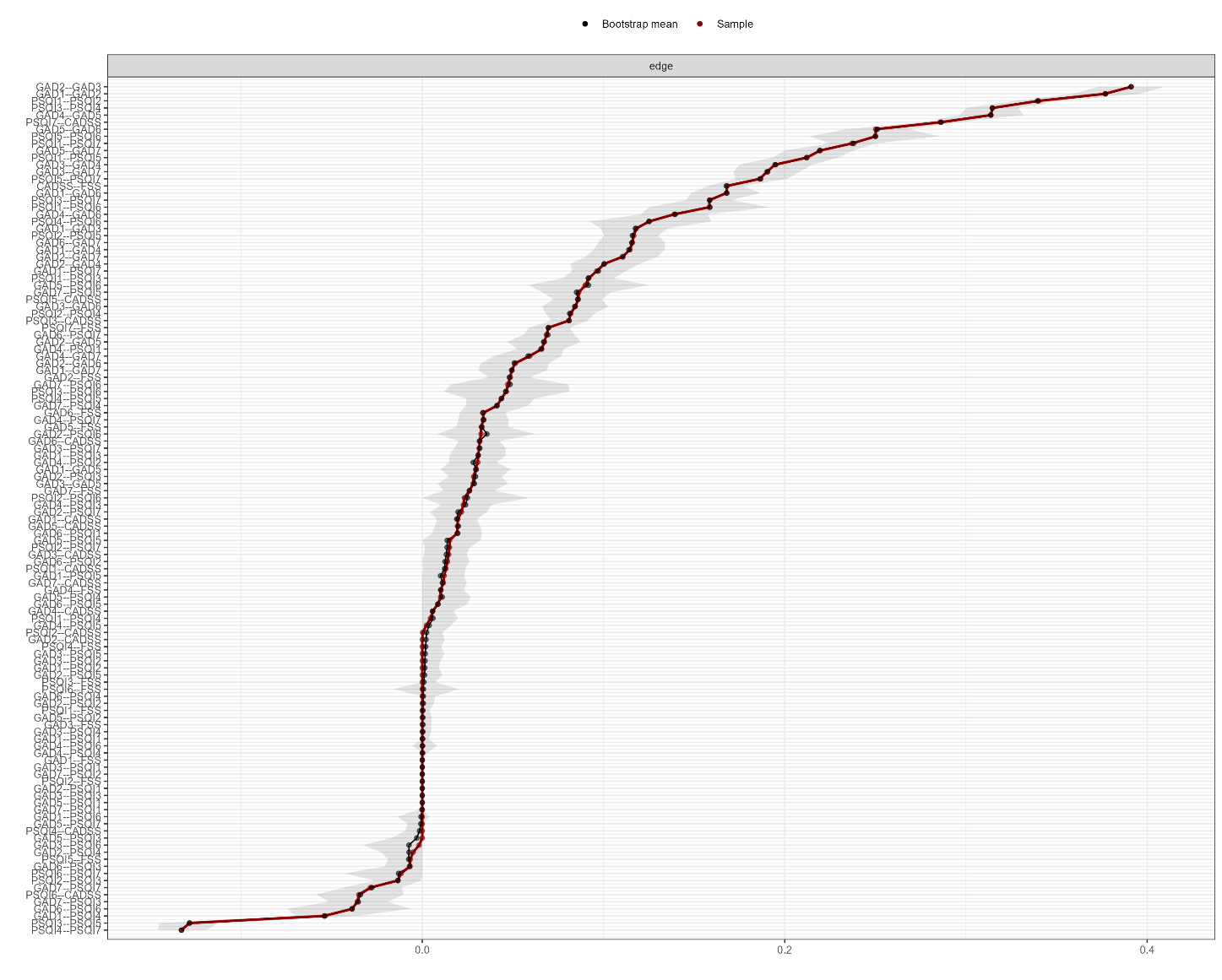


**Supplementary materials Figure 1**. Accuracy of edge weights.

Node: The red line represents the edge weight of the sample in this study, the black line represents the average edge weight evaluated by the self-help method, and the gray area represents the confidence interval obtained by the self-help method.

Note: GAD1: Nervousness, GAD2: Uncontrollable worrying, GAD3: Worry too much, GAD4: Trouble relaxing, GAD5: Restlessness, GAD6: Irritability; GAD7: Feeling afraid; PSQI1: Subjective sleep quality; PSQI2: Sleep latency; PSQI3: Sleep duration; PSQI4: Sleep efficiency; PSQI5: Sleep disturbance; PSQI6: Used sleep medication; PSQI7: Daytime dysfunction; CADSS: Chinese Adolescent Daytime Sleepiness Questionnaire; FSS: Fatigue Severity Scale.


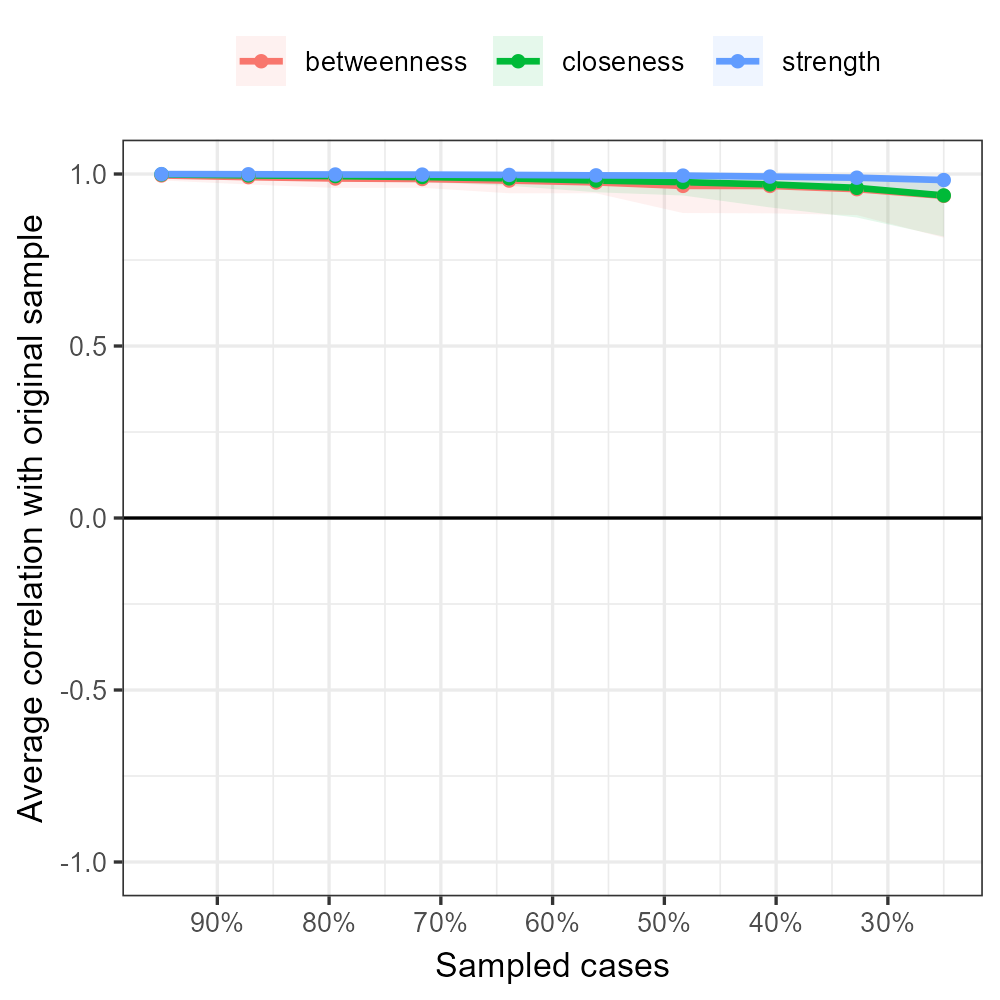


**Supplementary materials Figure 2**. Stability of centrality indices by case dropping subset bootstrap.

Note: The x-axis represents the percentage of cases of the original sample used at each step. The y-axis represents the average of correlations between the centrality indices in the original network and the centrality indices from the re-estimated networks after excluding increasing percentages of cases. The line indicates the correlations of strength and bridge strength.
