# Supplementary figures and images for: Network Analysis and Mediation Effect Analysis of Anxiety Symptoms and Sleep Patterns for Adolescents
Source: Actas Esp Psiquiatr. 2025 Oct 5;53(5):1040–52. doi: 10.62641/aep.v53i5.1961 (PMC12538618; doi:10.62641/aep.v53i5.1961)

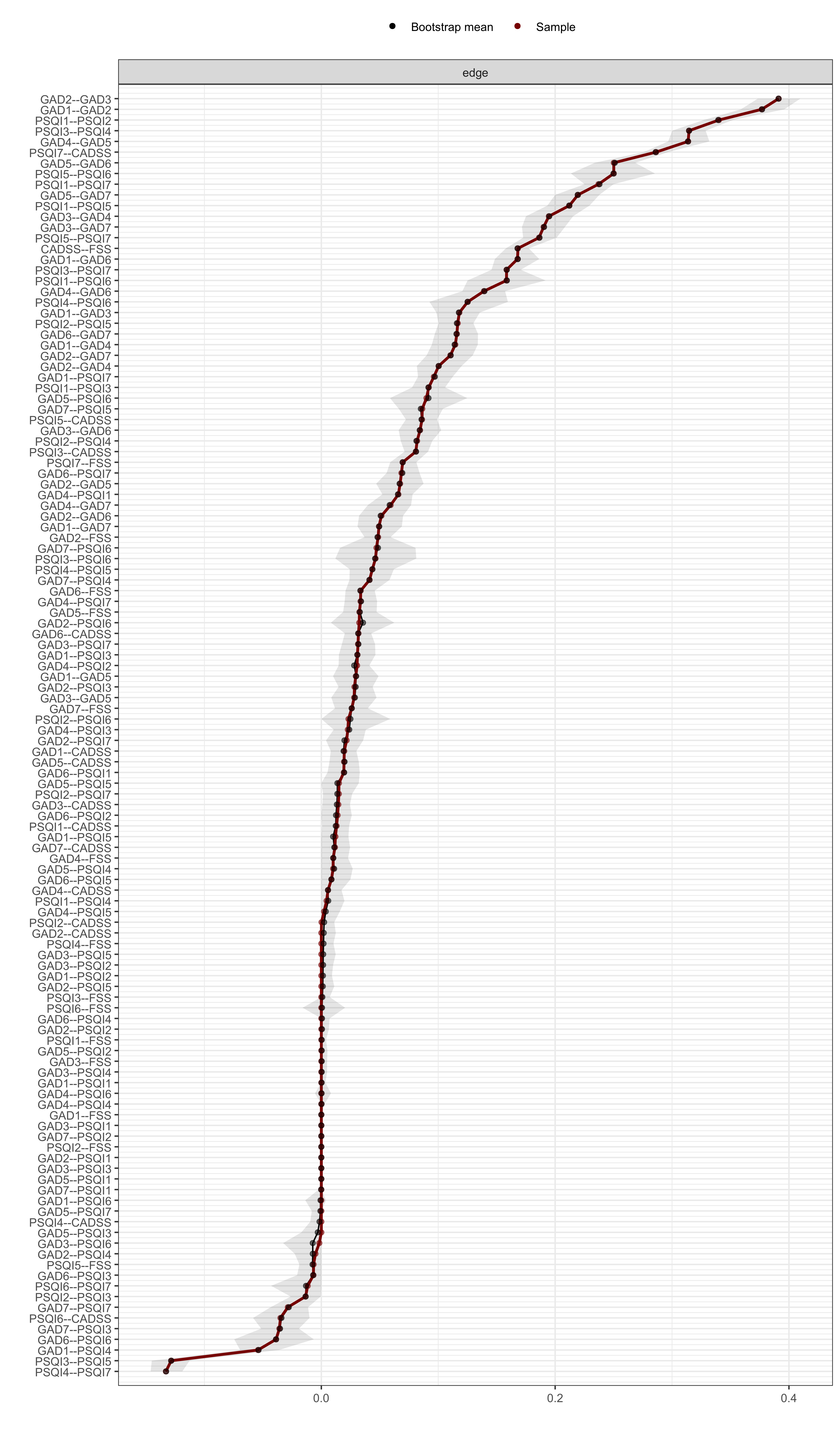

Supplement: Supplementary file 1 [file ActEsp-53-5-1040-1052-s1.zip › Supplementary Fig. 1.jpg]

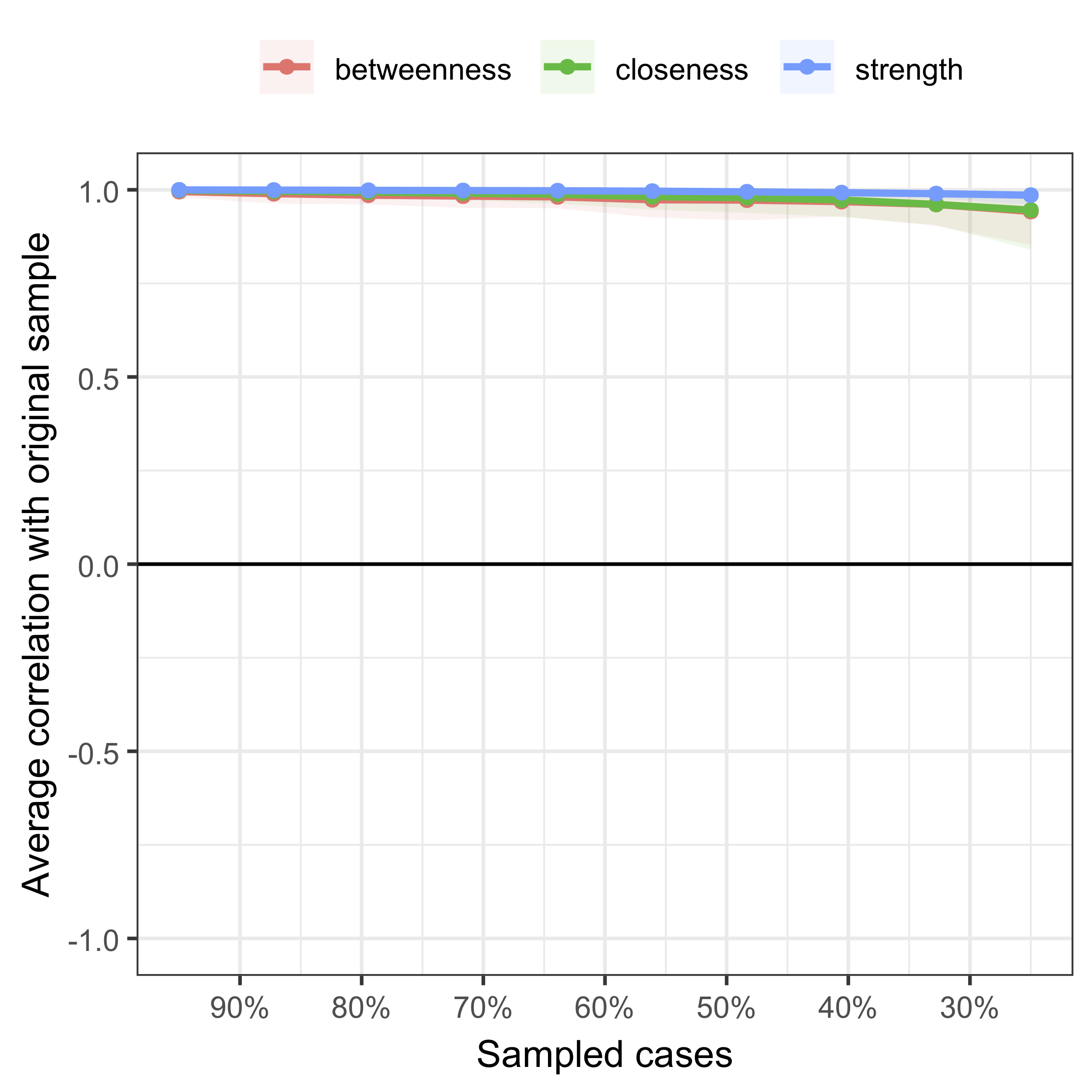

Supplement: Supplementary file 1 [file ActEsp-53-5-1040-1052-s1.zip › Supplementary Fig. 2.jpg]
